# Parental age and age gap in relation to neonatal health: a cohort study in eastern China

**DOI:** 10.3389/frph.2026.1818157

**Published:** 2026-06-22

**Authors:** Xuezhi Zhao, Siyuan Qian, Yongran Cheng, Weiguo Lu

**Affiliations:** 1 Department of Gynecology, Women's Hospital, School of Medicine, Zhejiang University, Hangzhou, China; 2 School of Laboratory Medicine and School of Bioengineering, Hangzhou Medical College, Hangzhou, China; 3 School of Public Health, Hangzhou Medical College, Hangzhou, China; 4 Zhejiang Key Laboratory of Maternal and Infant Health, Women's Hospital, School of Medicine, Zhejiang University, Hangzhou, China

**Keywords:** age gap, neonatal health, parental age, perinatal outcomes, retrospective cohort study

## Abstract

**Objective:**

To investigate the association between parental age, age gap, and neonatal health outcomes, and to provide epidemiological evidence for preconception counseling and perinatal risk assessment.

**Methods:**

This retrospective cohort study included mother–infant pairs who delivered at the Women's Hospital, Zhejiang University School of Medicine between January 2016 and December 2022. A total of 15,762 pairs were analyzed, comprising 7,557 healthy controls and 8,205 cases. The primary exposure was parental age comparison. Multivariable logistic regression was used to examine the association between parental age comparison and neonatal diseases, with adjustment for maternal BMI, hypertension, diabetes, gestational week, smoking, alcohol use, and educational level. Parental ages were also entered as continuous variables, and interaction terms were tested. Restricted cubic splines were applied to assess nonlinear dose–response relationships.

**Results:**

Compared with the equal-age group, both the father-older group (OR = 1.561, 95% CI: 1.203–3.201) and the mother-older group (OR = 1.652, 95% CI: 1.323–2.343) had significantly increased odds of neonatal diseases. After further adjustment for individual parental ages, the father-older association became non-significant (OR = 1.121, *P* = 0.420), while the mother-older association remained marginally significant (OR = 1.012, *P* = 0.022). Both maternal age (OR = 1.080, *P* < 0.001) and paternal age (OR = 1.051, *P* = 0.002) were independent risk factors. Significant interactions were observed between age gap and maternal age (OR = 1.098, *P* = 0.011) and between maternal and paternal ages (OR = 1.098, *P* = 0.032). Restricted cubic spline analysis showed that the risk of adverse neonatal outcomes accelerated after maternal age >30.1 years and paternal age >40.2 years (nonlinear *P* < 0.01 for both).

**Conclusion:**

Both maternal and paternal ages are independent risk factors with nonlinear dose-response relationships. The combined structure of parental ages should be considered in preconception counseling and perinatal risk assessment.

## Introduction

With increasing human life expectancy, rising societal pressures, and advances in medical technology, the age structure of childbearing among couples has undergone significant changes. The proportion of advanced maternal and paternal age has been progressively increasing (1). The International Federation of Gynecology and Obstetrics defines advanced maternal age as maternal age ≥35 years at the expected date of delivery, while no unified definition has been established for advanced paternal age (2, 3). The risks associated with advanced maternal age have been well-documented, including increased rates of miscarriage, pregnancy complications, and perinatal mortality, as well as reduced success rates of assisted reproductive technologies (4). Regarding advanced paternal age and pregnancy outcomes, several studies suggest that paternal age may affect sperm quality, miscarriage rates, and neonatal birth weight (5–9).

Although the individual effects of maternal and paternal age have been extensively explored, research on the impact of their age difference on neonatal health remains relatively limited. Existing studies concerning age disparity have primarily focused on how spousal age or age gap influences couple fertility (10, 11), rather than directly linking it to specific health outcomes. Currently, there is a scarcity of systematic, large-scale population-based evidence on how parental age difference specifically affects offspring health. A deeper understanding of the influence of parental age combination on child health holds significant implications for guiding family planning and clinical counseling.

Therefore, this study utilized a large contemporary birth cohort from eastern China to investigate the relationship between parental age structure and the burden of neonatal diseases. Our goal is to provide new epidemiological evidence to inform preconception counseling and early identification of life risks.

## Materials and methods

### Data source and study population

This study utilized anonymous medical records of newborns diagnosed with birth-related diseases at the Obstetrics and Gynecology Hospital of Zhejiang University School of Medicine from January 2016 to December 2022. The dataset included maternal age, paternal age, and neonatal disease diagnoses classified according to the International Classification of Diseases, 10th Edition (ICD-10). These diagnoses covered a wide range of conditions, and the list of disease categories can be found in Supplementary Table S1. The study covered cases of congenital abnormalities, metabolic disorders, premature birth, and respiratory diseases. After excluding invalid and erroneous samples, a total of 15,762 mother-child pairs were included, among which 7,557 were healthy controls and 8,205 were from the unhealthy group (patients with premature birth, metabolic disorders, respiratory diseases, or congenital abnormalities). This study followed the provisions of the Helsinki Declaration and was approved by the Ethics Committee of the Obstetrics and Gynecology Hospital of Zhejiang University School of Medicine (Approval Number: IRB-20240135-R). Since the data were retrospective and anonymous, informed consent was not required.

The primary outcome is any birth defect (comparison between the case group and the control group). The secondary outcomes are specific birth defects, including premature birth, metabolic disorders, respiratory diseases, and congenital abnormalities. The definition of cases is based on the classification criteria of the original dataset. The main exposure factor is the comparison of parental ages, divided into three groups: father's age greater than mother's (father > mother), mother's age greater than father's (father < mother), and parents' ages being equal (The “equal age” reference group was defined strictly as couples with an exact 0-year age difference, as this represents a clear demographic benchmark for reproductive age matching), with the “equal” group serving as the control group. The covariates include maternal body mass index (BMI, a continuous variable), hypertension (yes/no), diabetes (yes/no), gestational weeks (a continuous variable), maternal smoking during pregnancy (yes/no), alcohol consumption during pregnancy (yes/no) and Educational level. All binary covariates were coded with the absence of the condition as the reference value.

### Continuous parental age analysis

To explore the dose-response relationship, the ages of the mother and the father were also examined as continuous variables. Linear logistic regression models were applied to each parent's age separately and simultaneously. Additionally, a restricted cubic spline (RCS) with three knots (located at the 10th, 50th, and 90th percentiles) was used to consider possible nonlinear associations. Furthermore, different knot numbers were selected for sensitivity analysis. The RCS model was implemented using the rms package, while the OR values were plotted with the median age as the reference.

### Statistical analysis

Multivariable logistic regression models were constructed to estimate the association between parental age comparison and each outcome. All models were adjusted for the prespecified covariates: BMI, hypertension, diabetes, gestational week, smoking, and drinking. Results are reported as odds ratios (ORs) with 95% confidence intervals (CIs). For the primary outcome (any birth disease), both unadjusted and adjusted models were fitted. For each specific disease, a separate logistic regression model was built comparing cases of that disease with healthy controls.

All statistical analyses were performed using R software (version 4.2.3). Continuous variables are presented as mean ± standard deviation (SD) or median with interquartile range (IQR), and categorical variables as frequencies (percentages). Comparisons among groups were assessed using analysis of variance (ANOVA) for normally distributed continuous variables, the Kruskal–Wallis test for non-normally distributed continuous variables, and the chi-square test for categorical variables. A two-sided *P* < 0.05 was considered statistically significant.

## Results

### Study population and baseline characteristics

A total of 15,762 mother–infant pairs were included in the final analysis, comprising 7,557 healthy controls and 8,205 cases with at least one neonatal disease (preterm birth, metabolic disorder, respiratory disease, or congenital abnormality). As shown in Table 1, significant differences were observed between the control and disease groups across most baseline variables. Parents in the disease group were slightly older (father: 31.3 vs. 30.1 years; mother: 30.6 vs. 30.1 years), had higher maternal BMI (27.1 vs. 22.2 kg/m^2^), and shorter gestational weeks (34.0 vs. 39.5 weeks) compared with controls (all *P* < 0.001). Additionally, maternal hypertension, diabetes, smoking, and alcohol consumption during pregnancy were substantially more prevalent in the disease group (all *P* < 0.001). Educational level did not differ significantly between groups (*P* = 0.145).

**Table 1:** Baseline characteristics of participants.

Variable	Overall *N* = 15,762^a^	Control *N* = 7,557^a^	Disease *N* = 8,205^a^	*P*-value
Father's age (years)	30.7 (5.1)	30.1 (4.5)	31.3 (5.5)	<0.001
Mother's age (years)	30.3 (5.4)	30.1 (4.7)	30.6 (5.9)	<0.001
Maternal BMI (kg/m^2^)	24.7 (3.5)	22.2 (1.7)	27.1 (3.0)	<0.001
Gestational week	36.6 (3.7)	39.5 (1.1)	34.0 (3.2)	<0.001
Maternal hypertension	3,538 (22%)	528 (7.0%)	3,010 (37%)	<0.001
Maternal diabetes	3,041 (19%)	374 (4.9%)	2,667 (33%)	<0.001
Smoking during pregnancy	3,918 (25%)	658 (8.7%)	3,260 (40%)	<0.001
Drinking during pregnancy	3,549 (23%)	471 (6.2%)	3,078 (38%)	<0.001
Educational level				0.145
Junior college and below	5,108 (32.4%)	2,786 (36.9%)	2,322 (28.3%)	
Undergraduate degree	6,241 (39.6%)	2,864 (37.9%)	3,377 (41.2%)	
Graduate student	4,413 (28.0%)	1,907 (25.2%)	2,506 (30.5%)	

a Mean (SD); *N* (%).

### Association between parental age comparison and neonatal morbidity

Table 2 presents the multivariable logistic regression results for neonatal morbidity without adjusting for individual parental ages. Compared with couples of equal age, both the father-older group (OR = 1.561, 95% CI: 1.203–3.201, *P* < 0.001) and the mother-older group (OR = 1.652, 95% CI: 1.323–2.343, *P* < 0.001) were associated with significantly increased odds of any neonatal disease. Other significant independent risk factors included higher maternal BMI, maternal hypertension, diabetes, shorter gestational weeks, and maternal smoking or drinking during pregnancy (all *P* < 0.001).

**Table 2:** A multivariate logistic regression analysis was conducted on various factors affecting the health of newborns without adjusting for the ages of the parents.

Variables	OR	95% CI	*P*
Age compare (ref = equal)			
Father > mother	1.561	1.203–3.201	<0.001
Father < mother	1.652	1.323–2.343	<0.001
Maternal BMI (kg/m^2^)	1.169	1.111–1.341	<0.001
Maternal hypertension (ref = no)	1.250	1.096–2.214	<0.001
Maternal diabetes (ref = no)	1.245	1.112–1.657	<0.001
Gestational week	0.331	0.319–0.343	<0.001
Smoking during pregnancy (ref = no)	1.733	1.354–3.543	<0.001
Drinking during pregnancy (ref = no)	1.308	1.076–1.439	<0.001
Educational level (ref = junior college and below)			
Undergraduate degree	1.023	0.998–1.198	0.074
Graduate student	1.021	1.002–1.202	0.002

After further adjusting for parental ages as continuous variables (Table 3), the associations for the father-older group attenuated and became non-significant (OR = 1.121, 95% CI: 0.852–1.483, *P* = 0.420), while the mother-older group remained marginally significant (OR = 1.012, 95% CI: 1.009–1.031, *P* = 0.022). In this adjusted model, both increasing maternal age (OR = 1.080, 95% CI: 1.050–1.110, *P* < 0.001) and increasing paternal age (OR = 1.051, 95% CI: 1.020–1.083, *P* = 0.002) were independently associated with higher neonatal morbidity.

**Table 3:** A multivariate logistic regression analysis of various factors affecting the health of newborns, after adjusting for the ages of the parents.

Variables	OR	95% CI	*P*
Father age	1.051	1.020–1.083	0.002
Mother age	1.080	1.050–1.110	<0.001
Age compare (ref = equal)			
Father > mother	1.121	0.852–1.483	0.420
Father < mother	1.012	1.009–1.031	0.022
Maternal BMI (kg/m^2^)	1.122	1.081–1.166	<0.001
Maternal hypertension (ref = no)	1.182	1.021–1.364	0.030
Maternal diabetes (ref = no)	1.220	1.063–1.412	0.006
Gestational week	0.351	0.346–0.361	<0.001
Smoking during pregnancy (ref = no)	1.654	1.281–2.138	<0.001
Drinking during pregnancy (ref = no)	1.254	1.038–1.525	0.020
Educational level (ref = junior college and below)			
Undergraduate degree	1.042	0.938–1.108	0.063
Graduate student	1.018	1.006–1.132	0.003

### Interaction effects between parental ages

Table 4 shows the results of the multivariable logistic regression model examining interaction effects. Significant interactions were observed between age gap and maternal age (OR = 1.098, 95% CI: 1.072–1.129, *P* = 0.011) and between maternal and paternal ages (OR = 1.098, 95% CI: 1.011–1.148, *P* = 0.032). The interaction between age gap and paternal age did not reach statistical significance (OR = 1.198, 95% CI: 0.876–1.276, *P* = 0.061). These findings suggest that the effect of parental age disparity on neonatal health may be modified by the mother's age.

**Table 4:** A multivariate logistic regression analysis was conducted to examine the interaction effect of the ages of both parents on various factors influencing the health of newborns.

Variables	OR	95% CI	*P*
Father age	1.045	1.021–1.053	0.002
Mother age	1.083	1.051–1.191	<0.001
Age gap × mother age	1.098	1.072–1.129	0.011
Age gap × father age	1.198	0.876–1.276	0.061
Mother age × father age	1.098	1.011–1.148	0.032
Age compare(ref = equal)			
Father > mother	1.123	0.852–1.434	0.321
Father < mother	1.003	0.813–1.041	0.063
Maternal BMI (kg/m^2^)	1.110	1.061–1.131	<0.001
Maternal hypertension (ref = no)	1.132	1.011–1.204	0.025
Maternal diabetes (ref = no)	1.221	1.053–1.422	0.004
Gestational week	0.322	0.321–0.362	<0.001
Smoking during pregnancy(ref = no)	1.634	1.282–2.139	<0.001
Drinking during pregnancy(ref = no)	1.253	1.039–1.535	0.021
Educational level (ref = junior college and below)			
Undergraduate degree	1.022	0.934–1.128	0.053
Graduate student	1.012	1.004–1.102	0.004

### Continuous parental age analysis

**Figure 1:**
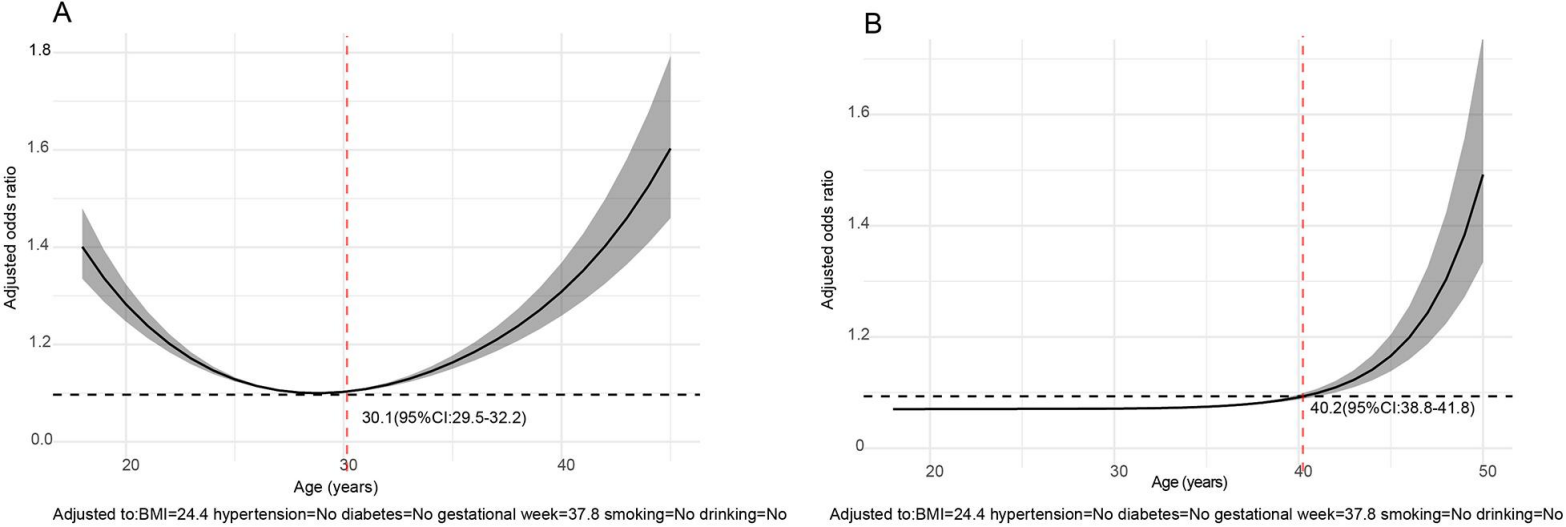
Adjusted odds ratios for neonatal morbidity according to parental age, using restricted cubic splines. The solid line represents the point estimate of the odds ratio, and the shaded area indicates the 95% confidence interval (Panel **(A)**: maternal age; Panel **(B)**: paternal age).

Figure 1 shows the adjusted dose-response relationship between parental age and neonatal morbidity, plotted using restricted cubic spline curves. For both mothers and fathers, the risk of adverse outcomes in the newborn increases gradually with age, and after the age of the mother's risk value (30.1 years old, 95% CI: 29.5–32.2) and the age of the father's risk value (40.2 years old, 95% CI: 38.8–41.8), the rate of this increase significantly accelerates. This non-linear trend is statistically significant for both parents (overall non-linear *P* value <0.01).

## Discussion

This large-scale cohort study of 15,762 mother-infant pairs in eastern China systematically explored the associations of parental age, spousal age gap, and their interactive effects with neonatal morbidity. The key findings demonstrated that compared with age-matched couples, parental age mismatch (either father-older or mother-older) was independently associated with elevated risks of adverse neonatal outcomes before adjustment; after full adjustment for individual parental age, the father-older group showed attenuated and non-significant associations, while the mother-older group remained marginally significant. Meanwhile, both advanced maternal and paternal age were independent risk factors for increased neonatal diseases, with a non-linear dose-response relationship—risk accelerated notably after maternal age of 30.1 years and paternal age of 40.2 years. Additionally, significant interactions existed between age gap and maternal age, as well as between maternal and paternal age, indicating that the impact of age disparity was modified by maternal age. These results supplement population-based evidence for the combined effects of parental age structure on neonatal health and provide empirical support for preconception counseling and perinatal risk assessment.

Consistent with previous epidemiological research, our study confirmed that advanced parental age was independently linked to higher neonatal morbidity. A growing body of evidence has validated that advanced maternal age (≥35 years) raises the risks of miscarriage, pregnancy complications, and adverse perinatal outcomes by reducing oocyte quality and increasing chromosomal aneuploidy (2, 4). For paternal age, studies have reported that advancing age impairs sperm DNA integrity, increases *de novo* mutations, and correlates with low birth weight, preterm birth, and congenital anomalies (5–7, 12). Our restricted cubic spline analysis further identified non-linear trends: neonatal adverse risk rose gradually with parental age and accelerated after specific thresholds, which aligns with age-related reproductive function decline, including diminished ovarian reserve, oocyte aneuploidy, and accumulated sperm DNA damage (13, 14). This non-linear association highlights the importance of age thresholds in reproductive health guidance, rather than a simple linear risk increment.

The findings of this study suggest that the age gap between parents may bring additional risks to the health of newborns beyond the factors related to individual age. Most of the existing research on the age difference between couples focuses on the aspect of childbearing (15, 16), while there is relatively less direct evidence based on the population linking the age gap to the health status of offspring. Potential mechanisms may include synchronous reproductive physiology, stable intrauterine environment, and consistent lifestyle habits of age-matched couples. A larger age gap may disrupt the epigenetic balance and increase the cumulative genetic risk of the fertilized egg (13), which may be attributed to the accumulation of new mutations and epigenetic changes in aging sperm (17, 18); and the influence of the mother's age being greater than that of the father is consistent with the known risks of egg quality and pregnancy complications (19).

Interaction analysis revealed that the effect of age gap on neonatal health was modified by maternal age, and a significant interaction also existed between maternal and paternal age. This suggests that the combined effect of parental age structure is not simply the superposition of individual age effects, but involves synergistic or regulatory mechanisms. A previous study also reported an interactive effect between paternal and maternal age on live birth rates in IVF/ICSI cycles (20), supporting the rationality of evaluating parental age as a combined structure. In clinical practice, this means that perinatal risk assessment should not only focus on individual parental age but also incorporate spousal age gap, especially for women of advanced age with a large age gap, who need enhanced monitoring.

From the perspective of reproductive health strategy, our findings support integrating parental age and age gap into routine preconception counseling and perinatal risk evaluation. Previous studies have recommended the optimal reproductive age for men as 25–35 years, with sperm genetic mutation risk doubling after 35 years (18–20), which is consistent with our observation of increased paternal risk after 40.2 years. For couples with large age gaps or advanced parental age, interventions such as enhanced genetic counseling, prenatal screening, and neonatal surveillance are recommended (21). Additionally, preconception health management for both parents—including paternal folate supplementation—may help reduce adverse neonatal risks (22). These implications are valuable for optimizing family planning and reducing the disease burden of birth defects.

However, this study has some limitations. Firstly, as a retrospective medical record study, this analysis is subject to potential information bias and residual confounding due to unmeasured or unavailable variables. Specifically, our hospital records did not contain information on important potential confounders such as maternal history of previous adverse pregnancy outcomes (e.g., stillbirth, preterm birth), severe iron and folate deficiency anaemia, parental or family genetic disorders (e.g., sickle cell anaemia, thalassemia), rubella infection status during pregnancy, or preconception care utilization. The absence of these factors may have led to residual confounding, and the observed associations between parental age structure and neonatal morbidity could be partially attributed to or modified by these unmeasured variables. Future prospective studies should collect detailed information on these factors to strengthen causal inference. Secondly, the sample comes from a tertiary hospital in eastern China, which may limit its general applicability in other regions and ethnic groups. Thirdly, the study was conducted at a single tertiary care center that likely overrepresents high-risk pregnancies, so the findings may not apply to the general population or other regions/ethnic groups. Finally, the biological mechanism between the parental age gap and neonatal health remains unclear and requires further experimental verification.

## Conclusion

In conclusion, parental age and age gap jointly affect neonatal health, with advanced parental age associated with increased neonatal morbidity. The non-linear age-risk relationship and maternal age-modified age gap effect provide precise evidence for reproductive health guidance. Future research should adopt prospective designs and multi-center samples to verify the generalizability of the results and explore the underlying biological mechanisms, so as to provide a more scientific basis for reducing birth-related adverse outcome.

## Data Availability

The original data presented in the study are includedin the article/supplementary material, further inquiries can bedirected to the corresponding authors.
